# Alternative Promoters Influence Alternative Splicing at the Genomic Level

**DOI:** 10.1371/journal.pone.0002377

**Published:** 2008-06-18

**Authors:** Dedong Xin, Landian Hu, Xiangyin Kong

**Affiliations:** 1 Institute of Health Sciences, Shanghai Institutes for Biological Sciences, Chinese Academy of Sciences, Shanghai, People's Republic of China; 2 Ruijin Hospital, Shanghai Jiao Tong University School of Medicine, Shanghai, People's Republic of China; 3 State Key Laboratory of Medical Genomics, Ruijin Hospital, Shanghai Jiao Tong University School of Medicine, Shanghai, People's Republic of China; Centre de Regulació Genòmica, Spain

## Abstract

**Background:**

More and more experiments have shown that transcription and mRNA processing are not two independent events but are tightly coupled to each other. Both promoter and transcription rate were found to influence alternative splicing. More than half of human genes have alternative promoters, but it is still not clear why there are so many alternative promoters and what their biological roles are.

**Methodology/Principal Findings:**

In this study, we explored whether there is a functional correlation between alternative promoters and alternative splicing by a genome-wide analysis of human and mouse genes. We constructed a large data set of genes with alternative promoter and alternative splicing annotations. By analyzing these genes, we showed that genes with alternative promoters tended to demonstrate alternative splicing compare to genes with single promoter, and, genes with more alternative promoters tend to have more alternative splicing variants. Furthermore, transcripts from different alternative promoters tended to splice differently.

**Conclusions/Significance:**

Thus at the genomic level, alternative promoters are positively correlated with alternative splicing.

## Introduction

Alternative splicing (AS) is a common post-transcriptional process used by eukaryotic organisms to generate multiple transcript variants from a single gene [1]. It is known that approximately 60% of human genes are susceptible to AS [2], [3]. Substantial evidence has indicated that AS plays an important role in development, differentiation, and cancer [4]–[6], and misregulation of AS is associated with human disease [7]. AS is also considered as the major mechanism expanding protein diversity.

Most research on the regulation of AS has focused on identifying sequence features (cis-acting elements) within the mRNA that either enhance or silence the usage of adjacent splice sites, and the proteins that bind to these sequences (trans-acting elements) to define global splicing patterns. The known cis-acting sequence elements in exons and introns that are important for promoting splice-site recognition include exonic splicing enhancers (ESEs), intronic splicing enhancers (ISEs), exonic splicing silencers (ESSs), and intronic splicing silencers (ISSs) [8]. These sequences tend to be short (typically ∼5–10 nt in length) and consist of relatively degenerate consensus sequences that are recognized by trans-acting splicing factors, such as serine/arginine-rich domain proteins (SR proteins) and nuclear ribonucleoprotein proteins (hnRNPs), which can regulate the use of a splice site [8]–[10].

Besides the cis-acting and trans-acting factors identified above, a number of studies have revealed that promoters may also be involved in the regulation of pre-mRNA splicing [11]. When an alternatively spliced exon (EDA) from the fibronectin 1 gene was chimerized with alpha 1 hemoglobin gene and formed a globin-fibronectin gene, the ratio of mRNA that included or excluded this alternative exon was shown to be strongly influenced by the promoter used to drive transcription [12], [13]. The cytomegalovirus promoter resulted in an EDA inclusion several times more than that of alpha-globin [12], [13]. Similar effects of promoters on AS have also been found in other genes [14]–[16].

It was revealed that more than half of human genes (at least 53%) have alternative promoters (APs) [17]. A number of studies indicated that APs of a gene have different tissue specificity [18], [19], developmental activity [20] and/or transcription activity [21], and generate transcripts with different 5′ untranslated regions (5′UTR) or open reading frames (ORF) [22]. But it is still not clear why there are so many APs in genome. Different APs may be different in promoter elements such as CpG islands [17]. Thus it is possible that genes with APs have the ability to generate AS variants. There are also several cases suggesting that APs are linked to AS [23], [24], but whether this happens only in experimental conditions or universally exist at the genomic level in physiological condition is not known.

In this paper, we have extracted and combined APs and AS annotations from several databases and demonstrated that genes with APs tended to generate AS variants. Additionally, the number of APs was positively correlated with the number of AS variants within the genes with APs and AS variants. Comparing the mouse full-length cDNA sequences further demonstrated that two transcripts transcribed by different APs tended to splice differently. We concluded that at the genomic level, APs are positive correlated with AS.

## Materials and Methods

### Identification AS and non-AS genes

AS annotations of human and mouse genes were obtained from AltSplice database (http://www.ebi.ac.uk/asd/) and Ecgene (http://genome.ewha.ac.kr/ECgene/). The splice annotation data in AltSplice database was generated by comparison of EST/mRNA mapping data. Transcripts that have the same exon structures were grouped into ‘class’. In principle, different ‘classes’ represented different splice patterns. Ecgene used an algorithm and EST/mRNA resource which was different from AltSplice. The AS annotations we used in this study were based on the annotations from AltSplice and Ecgene databases. 16560 human and 15986 mouse annotated genes were extracted from the AltSplice version 3 dataset. Another set of genes with AS annotations were extracted from the Ecgene dataset. Ecgene IDs were converted to Ensembl gene IDs, such that annotations in the Ecgene dataset could be merged with that of the AltSplice dataset (see below). We obtained 8644 human and 8670 mouse genes with annotations of AS from Ecgene dataset.

Genes with AS variants identified in both AltSplice and Ecgene datasets were defined as alternatively spliced genes. Genes with no AS variants identified in both datasets were defined as non-alternatively spliced genes.

### Estimation of alternatively spliced variants

We used the ‘class’ number extracted from the AltSplice database as the estimate of the number of AS variants. But there were two situations we considered with caution. Firstly, there may have been classes that did not overlap with one another. The second was although two classes overlapped with each other, the region of overlap may have had the same structure. The classes in these two situations may not have represented a distinct splice variant (see http://www.ebi.ac.uk/asd/altsplice/AltSplice-pipeline.pdf). Thus, besides using ‘class’ number as the estimate of splice variant number, we have also used a weighted method such that a class was counted as 0.5 splice variant if it followed the above two situations.

As an alternative to the AltSplice data, the number of splicing variants in the Ecgene database was calculated using a graph-theory based algorithm [25]. The exon and intron structure of a gene were presented as a directed acyclic graph (DAG). Nodes and edges correspond to exons and introns respectively and each possible path in the DAG corresponded to a putative splice variant. Then the EST and/or mRNA (from Unigene) supported splice variants were searched. We only counted the splice variants verified by at least one full-length clone as our estimate of AS for the purpose of reliability.

### Identifying alternative promoter numbers

AP annotations of human and mouse genes were extracted from the dataset downloaded from Database of Transcriptional Start Sites (DBTSS). Refseq IDs in the dataset were converted into Ensembl gene IDs, so as to merge the AP annotations with AS annotations by their common Ensembl gene IDs. Some genes (79 for human and 31 for mouse) were removed from our dataset due to ambiguous mapping of their IDs. Finally we obtained 14036 and 13134 human and mouse gene datasets with AP annotations, respectively.

### Analysis of the relationship of AP and AS in full-length cDNA data

Full-length cDNA genome mapping data was downloaded from the FANTOM3 FTP site (http://fantom.gsc.riken.go.jp/download.html). There were 102801 full-length enriched sequences (including full-length and partial sequences) in the Fantom3 dataset. All of these sequences were mapped to the mouse genome and annotated by the Fantom3 annotation system [26], [27]. Any transcript that mapped to the same chromosomal strand and shared at least one exonic nucleotide overlap was grouped into a transcription unit (TU) [27]. To ensure the sequences we used were full-length protein-coding cDNA, we used several steps to filter the original dataset:

- Eliminate the sequences from other resources (e.g. GenBank) and only the sequences from RIKEN and Refseq are kept in out dataset.
- Discard the sequences without putative 5′UTR and 3′UTR annotations.
- Remove the sequences that have no confirmed strand annotations.

Finally, 26715 full-length cDNA mapping data were left. After we eliminated the TUs with only one cDNA sequence, we finally obtained 8807 TUs with at least two reliable full-length cDNA sequences.

**Figure 1:**
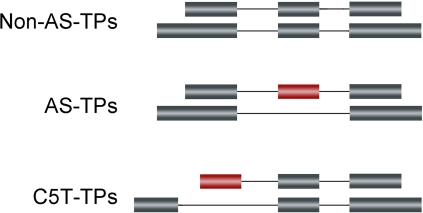
Classification of transcript pairs (TPs) based on the AS events derived from exon coordinates comparison. In all three examples of full-length cDNA comparisons, constitutive exons are shown in gray, alternatively spliced exons are shown in red, and introns are represented by solid lines. The first exon and last exon in each example represents the 5′ most exons and the 3′ most exons of full-length cDNA, respectively. Non-AS-TPs: TPs with members that are not alternatively spliced. As shown in the figure, differences in exterior part of the 5′ most or 3′ most exons are not considered an AS event. AS-TPs: TPs with members that are alternatively spliced. Any difference in the interior exon or the interior part of the 5′ most or 3′ most exons is considered an AS event. The figure shows an exon skipping event, used to represent other AS events including exon skipping, alternative 5′ splice sites, alternative 3′ splice sites, intron retention, and mutually exclusive. C5T-TPs: TPs were classified as C5T-TPs in cases in which the 5′ most exon of one transcript is completely mapped to the intronic region of another transcript, and there is no other type of AS event.

For each TU, all possible transcript combinations, or transcript pairs (TPs), were collected. To investigate whether two transcripts in a TP are alternatively spliced, an algorithm was developed. Basically, given a TP, we compared the exon coordinates of one member with those of the other member. An AS event was defined by the exon coordinates comparison status. There were three states of comparison between an exon and a transcript: falling outside the coordinates of the transcript, overlapping with an exon of the transcript, and completely falling inside an intronic region. Exons with coordinates that fell outside of the transcripts were not informative, thus we only considered the last two states in the following analyses. For an internal exon, an AS event was considered as either completely mapping within the intronic region or overlapping with the exon but extending or truncating at one or both ends. For a 5′ or 3′ terminal exon with overlapping status, only extending or truncating at the interior end was considered an AS event. For a 3′ terminal exon, if it was mapped completely to the intronic region of another transcript, an AS event was considered. TPs with one or more AS events mentioned above were considered alternatively spliced and grouped as AS-TPs. There was another AS event, which was derived from a situation in which the 5′ terminal exon was completely mapped to intronic region of another transcript. We treated this type of AS event separately, and TPs with only this AS event were classified into another TP group named C5T-TPs. The reason for this was that our intention was to assess the hypothesis that APs have regulatory effects on AS, whereas this type of AS event is definitely correlated with TSS, and thus not informative. TPs without any of the AS events were grouped as Non-AS-TPs (Figure 1).

Two transcripts with a TSS distance interval larger than 500 bp were considered to be transcribed from different APs. The criterion was believed to be strict enough to ensure that the transcripts were transcribed from different promoters [17].

All the statistic analyses were conducted by R.

## Results

### Genes with APs tend to show splicing

The idea that transcription and pre-mRNA processing are independent events has dominated our conception of pre-mRNA processing for a long time. But recently, evidence has indicated that transcription and splicing are tightly coupled to each other [28]. Transcripts from different promoters lead to changes in exon inclusion levels or AS pattern [12], [13], [15]. Possible mechanisms include the recruitment of factors with dual functions in transcription and splicing, and control of RNA polymerase II elongation [28].

52% of human and 27.8% of mouse genes have APs in the DBTSS. APs in the same gene may contain different promoter elements such as CpG islands [17]. Thus genes with APs possibly generate AS variants. There is also experimental evidence suggesting that APs are linked to AS [23], [29]. It is highly intriguing to examine whether genes with APs tend to demonstrate AS at the genomic level.

We obtained 7909 human genes and 7602 mouse genes with AS and AP annotations from the AlterSplice and Ecgene datasets. 6728 out of 7909 human genes and 5698 out of 7602 mouse genes were identified as alternatively spliced genes (Table 1: Alternatively spliced genes) which have AS variants in both databases (see Material and methods). The remaining 1181 human genes and 1904 mouse genes (Table 1: Others) were those that have no AS variants in one or both of the databases.

**Table 1:** The percentage of alternatively spliced genes among groups of single-promoter genes and multiple-promoter genes.

	Human		Mouse	
	Alternatively spliced genes (Percentage)	Others (Percentage)	Alternatively spliced genes (Percentage)	Others (Percentage)
Single-promoter genes	2692 (76.5%)	827 (23.5%)	3744 (69.7%)	1625 (30.3%)
Multiple-promoter genes	4036 (91.9%)	354 (8.1%)	1954 (87.5%)	279 (12.5%)

Note. The percentage alternatively spliced genes is different between groups of single-promoter genes and of multiple-promoter genes (P<0.001 by *x* ^2^ Test). Alternatively spliced genes: genes that have AS variants in both AlterSplice and Ecgene datasets. Others: genes that have no AS variants in one or both of the datasets.

We then compared the percentages of alternatively spliced genes in genes with APs (multiple-promoter genes) or genes without APs (single-promoter genes). Our results showed that multiple-promoter genes were notably enriched with alternatively spliced genes. As shown in Table 1, among multiple-promoter genes, we observed 91.9% of human and 87.5% of mouse genes were alternatively spliced genes. However, among single-promoter genes, we only observed 76.5% of human and 69.7% of mouse genes generating AS variants. Our results indicated that compared with single-promoter genes, multiple-promoter genes tended to demonstrate AS. However, more than half of human and mouse single-promoter genes also had AS variants. This indicated that APs may be not indispensable for the generation of AS. Another possibility was that the single-promoter genes were indeed multiple-promoter genes, but mis-annotated as single-promoter genes due to lack of full-length cDNA data.

Conversely, do genes that have no AS variants tend to have single promoters? We have also identified 250 human and 508 mouse non-alternatively spliced genes (see Materials and Methods for the definition). As expected, among the non-alternatively spliced genes, we observed that 215 human and 479 mouse genes have single promoters (Table 2), which were significantly higher than as expected due to chance alone (p<0.001, Hyper-geometric Test).

**Table 2:** The number of genes with single promoter among non-alternatively spliced genes.

	All	Genes with single promoter	P-values
Human	250	215	1.509555e-44
Mouse	508	479	3.023425e-44

Note. The vast majority of the non-alternatively spliced genes have a single promoter. All: the number of all the non-alternatively spliced genes. Genes with single promoter: the number of genes with single promoter among non-alternatively spliced genes. P-value: the probability that an observation (non-alternatively spliced genes with single promoter) is made by chance, is calculated using the cumulative Hyper-geometric Distribution.

### Positive relationship between the number of APs and the number of AS variants

Our above results suggest that genes with APs are more likely to demonstrate AS. We then further examined whether there is a positive relationship between the number of AP and the number of AS variants among the multiple-promoter genes with AS variants (MPAS genes). We first classified human and mouse MPAS genes into three groups based on the AP numbers (2∼3, 4∼5, 6∼), and then calculated and compared the mean number of AS variants among the genes in each group.

**Figure 2:**
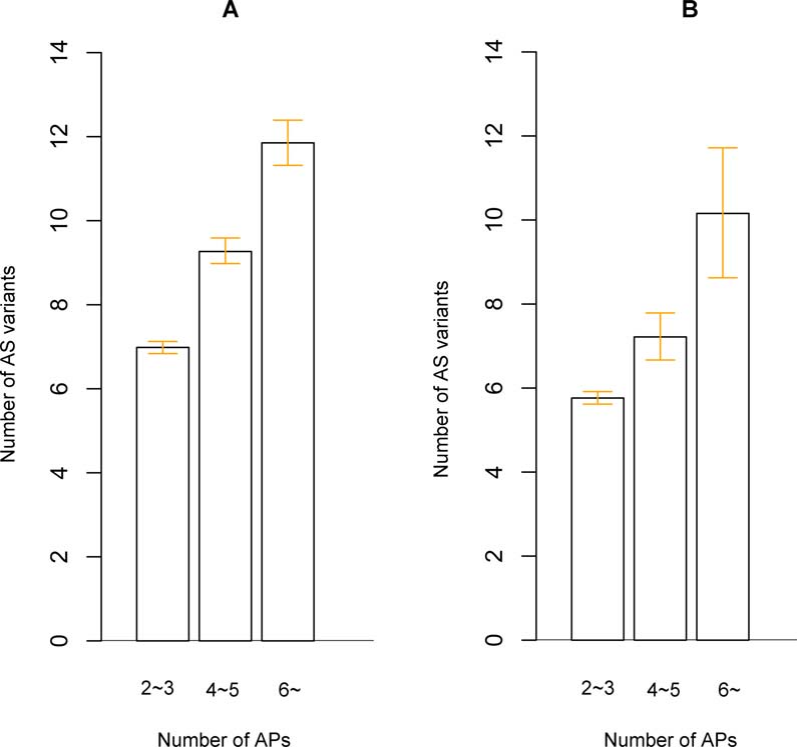
Genes with more APs tend to generate more AS variants. Number of AS variants was calculated based on AlterSplice dataset.” Genes with more APs showed an increased mean AS variants in human (A) and mouse (B) genes with APs and AS variants (MPAS genes). Error bar, 95% confidence intervals obtained from nonparametric bootstrapping.

As shown in Figure 2, human and mouse MPAS genes were classified into three groups according to the AP numbers (with a bin of 2∼3, 4∼5, 6∼). The mean number of AS variants for human genes with 2∼3 promoters and 4∼5 promoters were 6.98 and 9.28, respectively. However the mean number of AS variants for genes with 6∼ promoters was 11.85,nearly doubled the genes with 2∼3 promoters (p<0.001, Wilcoxon Test) (Figure 2A).

Similar results were observed in mouse MPAS genes (Figure 2B). The mean number of AS variants of mouse genes was 5.76 for those with 2∼3 promoters, 7.22 for those with 4∼5 promoters, however the mean number of AS variants increased to 10.16 for genes with 6∼ promoters (p<0.001 for genes with 2∼3 promoters versus genes with 4∼5 promoters; p<0.001 for genes with 4∼5 promoters versus those with 6∼ promoters; Wilcoxon Test).

Overall, the above results suggest that at the genomic level, a positive correlation exists between the number of APs and the number of AS variants among MPAS genes. Genes with more APs tend to generate more AS variants.

We used ‘class’ numbers extracted from AltSplic to represent the AS variant numbers in the above analysis. However, ‘class’ numbers may not represent AS numbers in the following two situations. In one case, a ‘class’ which does not overlap with another ‘class’ may represent different regions of the gene. The second is the case in which the overlapped region of the two ‘classes’ has the same exon structure (see http://www.ebi.ac.uk/asd/altsplice/AltSplice-pipeline.pdf). We recalculated the number AS variants using a new method, which gave these ‘classes’ a smaller weight. That is, if two ‘classes’ have no overlap or the overlapped region with the same exon structure, we counted each ‘class’ as 0.5 AS variant. The analysis using the new AS variant number revealed that although the mean AS variants for each AP group was less than those of our previous analysis, genes with more APs always had a higher number of mean AS variants (Supplementary Figure S1).

We have found that the number of APs was also positively correlated with the number of exon per gene (Supplementary Figure S2). To verify whether the correlation influenced our results, we grouped genes with similar exon numbers, and examined the correlation of number of APs and number of AS variants in each groups. Our results indicated that the positive correlation between AP and AS presented over all the intervals of exon count (Supplementary Figure S3). Since numbers of APs have no correlation with EST coverage per exon (Supplementary Figure S4), we can also eliminate the possibility that our results were due to the correlation of APs with the number of covered ESTs per exon.

**Figure 3:**
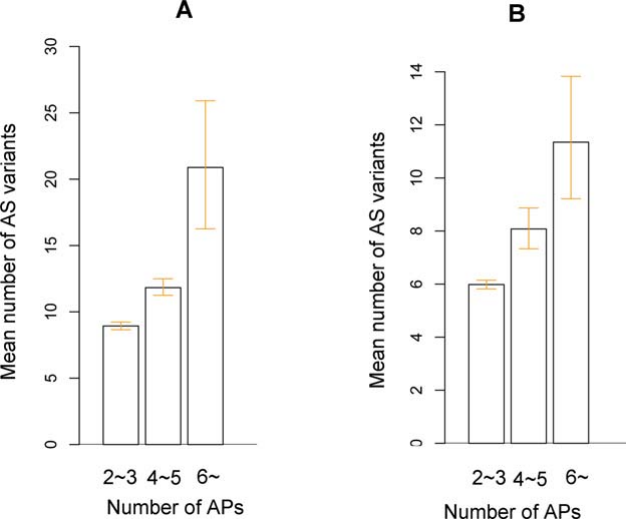
Positive relationship between the number of APs and the number of AS variants calculated using Ecgene dataset. Genes with more APs had more AS variants in both human (A) and mouse genes (B). Error bar, 95% confidence intervals obtained from nonparametric bootstrapping.

We repeated our analysis using the dataset extracted from Ecgene database, which use EST/mRNA resource and algorithm different from AltSplice database. The results were essentially identical to those from the AltSplice dataset (Figure 3).

Overall, this part of analyses revealed that among MPAS genes, the number of APs is positively correlated with the number of AS forms at the genomic level, which is consistent with experimental results showing that promoter structure can affect AS [28].

### Transcripts from different alternative promoters tend to splice differently

About 103000 entirely sequenced cDNA clones in the FANTOM3 database from the RIKEN mouse Gene Encyclopedia project facilitated the direct testing of whether transcripts from different APs tended to splice differently for a given gene. In the FANTOM3 data set, transcripts from several sources (e.g. GenBank and Ensembl) have been collected and mapped to the mouse genome. Sequences that contain common core genetic information (in some cases, a protein-coding region) were clustered into TUs [27]. We used several eliminating steps to guarantee the sequences we used were indeed full-length cDNA (see Materials and Methods). Finally, we obtained 26715 full-length cDNAs belonging to 8807 TUs.

Two transcripts with same TSS were considered as the same transcript and TPs composed of such transcripts were discarded. After filtering out 12750 TPs composed of same transcripts, 51578 pairs composed of putatively different transcripts remained.

To determine whether two transcripts of a TP were transcribed from different APs, we measured and compared their TSS distance. TPs with a TSS distance larger than 500 bp were considered to be transcribed from different APs, and conversely TPs with a TSS distance smaller than 500 bp were considered to be transcribed from the same AP. This finally yielded 5927 TPs which might be transcribed from different APs (“TPs with different APs”) and 45651 TPs which might be transcribed from the same APs (“TPs with same APs”).

Whether members of a TP were alternatively spliced was determined by comparison of the exon genomic coordinates of each transcript. TPs were divided into three groups based on the AS event derived from the exon coordinate comparison (see Materials and Methods). TPs were grouped as Non-AS-TPs if transcripts were not alternatively spliced. Others TPs with alternatively spliced transcripts were either grouped as AS-TPs or C5T-TPs (Figure 1).

**Figure 4:**
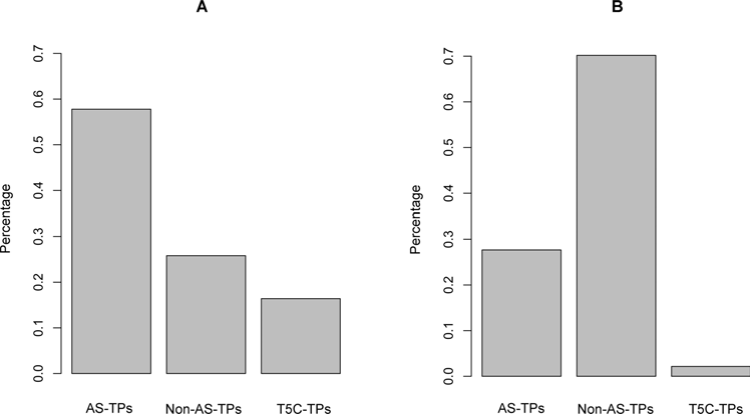
Percentage distributions of “AS-TPs”, “Non-AS-TPs” and “C5T-TPs” across “TPs with different APs” and “TPs with same APs”. (A) The distributions of TP percentages in “TPs with different APs” group. The percentage of AS-TP (57.8%) is two times larger than that of the Non-AS-TPs (25.8%). The proportion of C5T-TPs is 16.4%. (B) The distribution of TP percentages in “TPs with same APs”. The percentage of Non-AS-TPs (70%) is notably larger than that of AS-TPs (27.7%). The remaining 2.2% belongs to C5T-TPs.

To test whether transcripts transcribed from different APs tended to be alternatively spliced, we examined the percentage distributions of AS-TPs and Non-AS-TPs in the “TPs with different APs” and “TPs with same APs” groups, respectively. As indicated in Figure 4A, among “TPs with different APs”, the percentage of AS-TPs was approximately 57.8% (3425/5927), while that of Non-AS-TPs was approximately 25.8% (1530/5927). The percentage of AS-TPs was double that of Non-AS-TPs. Thus, two transcripts tended to be alternatively spliced if they were transcribed from different APs. Since “AS-TPs” mentioned above did not include “C5T-TPs” (representing approximately 16.4% all “TPs with different Aps”), our result implied that the positive correlation between AP and AS might be resulted from regulatory effect of AP on AS.

By analyzing the “TPs with same APs”, we found a reverse distribution in the frequencies of AS-TPs and Non-AS-TPs compared to “TPs with different APs”. As shown in Figure 4B, the majority of TPs were Non-AS-TP (70%, 32035/45651), compared to AS_TPs (27.7%, 12631/45651). Of the remainder, 2.2% were C5T-APs. The data suggested that transcripts with the same APs are not likely alternatively spliced, consistent with the analysis of “TP with different APs”.

The differences in the percentage of AS-TPs and Non-AS-TPs in “TPs with same APs” compared with “TPs with different APs” was statistically significant (P<0.001 for both comparison, *x* ^2^ Test).

We also divided the TPs into multiple groups according to the TSS distance interval, and recalculated the percentages of “AS-TPs”, “Non-AS-TPs” and “C5T-TPs” in each group. We observed that the distribution bias towards “AS-TPs” in groups with TSS distance spans of “300∼500 bp”, “500∼1000 bp” and “1000∼bp” (see Supplementary Figure S5). This result indicated that splice patterns tended to be different if two transcripts have TSS distances larger than 300 bp. Thus, our above result was independent of the criterion (500 bp interval) we used to divide the TPs.

In total, our analyses revealed that transcripts transcribed from different APs tended to be alternatively spliced and thus provided additional support for the relationship between APs and AS.

## Discussion

In this study, we conducted genome-wide studies of the relationship between APs and AS using the data collected from publicly available resources. Our results revealed that genes with APs are more likely to generate AS variants than genes with a single promoter. Interestingly, genes that do not undergo AS were significantly enriched within single-promoter genes. Thus, it seems that the AS variants of a gene are highly correlated with the AP number it possesses. We have found that the number of AP was positively correlated with the number of AS variants in genes with APs generating AS variants. By comparing full-length cDNA mapping data in each TU, we have also found direct evidence to show that transcripts transcribed from different APs tended to be alternatively spliced.

In our analysis, parts of AS annotations were extracted from AltSplice dataset, in which transcripts with different splicing pattern were grouped into separate ‘classes’. However, in some situations, the different ‘classes’ may not have represented different AS variants, but different regions of the genes. Thus, using the ‘classes’ number as the estimate of the number of AS variants may overestimate the genuine number of AS variants. We have used a weighted method to reduce such bias in case ‘classes’ did not represent genuine AS variants. Our results showed, although the mean number of AS variants was reduced relative to the former method, the positive relationship was still present across all ranges of APs. Using the AS variant numbers estimated from the Ecgene dataset also supported the positive relationship between the number of APs and the number of AS variants.

Our finding is in consistent with the recent finding that transcription is involved in mRNA processing. The most supportive evidence showing that transcription and AS are coupled with each other is that the use of different promoters resulted in drastic changes in an alternatively spliced exon inclusion [12], [13]. This promoter-dependent AS pattern has also been found in other experiments [14]–[16]. Several splicing proteins have been shown to interact directly with the C-terminal domain (CTD) of Pol II [30]–[32]. A recruitment model was raised to describe these results [28], [33]. Binding of splicing factors to the CTD increases their local concentration, thereby promoting otherwise weak interactions between the splicing factors and the pre-mRNA.

More and more studies have suggested that RNA polymerase II (Pol II) elongation can affect the frequency of exon inclusion. Low Pol II elongation rates would favor the inclusion of alternative exons, whereas a high elongation rate would favor exclusion of these exons [34]–[36]. These results were described by a kinetic model [22], [28]. This model states that slowing Pol II transcriptional elongation allows spliceosomal components to bind to the weak exon without having to compete with a subsequent strong exon, therefore favoring the recognition and inclusion of the weak exon. Faster elongation results in skipping of the weak exons because the subsequent strong exons compete with the spliceosomal components for the weak exons. Our findings that APs are positively correlated with AS was consistent with the finding that promoter structure affects AS and the above two models. Different APs could recruit promoter specific transcription co-activator and splicing proteins, which would in turn influence the Pol II elongation rate. Both the specificity of the splicing proteins and the Pol II elongation rates lead to different splicing. Other factors, such as difference in 5′UTRs of transcripts from different APs, may also contribute to the AS.

Alternative promoters allow gene expression at different times and in different tissues. Indeed, we found genes that with APs are expressed more broadly in tissues than those with single promoters (Supplementary Figure S6). Thus transcripts from different APs have the opportunity to present in different tissue environments and are regulated by tissue specific splicing factors and other trans-acting factors that are involved in AS. This might be one reason that in genomic level APs are positive correlated with AS. Correspondingly, beneficial alternative splicing might in turn promote the fix of an alternative promoter. Therefore, as a result of co-evolution of APs and AS, genes with more APs tend to have more AS variants.

AS is an important mechanism by which genes obtain function diversity. Different promoters respond to different environments, and regulate factors to function with appropriate splicing variants. The evolutionary conserved positive correlation between APs and AS probably facilitates quick adaptation of a species to a changed environment.

## Supporting Information

Figure S1 Positive relationship between the number of APs and the number of AS variants calculated by a weighted method in APAS genes. (A) The mean number of AS variants for each AP group of human APAS genes. The number of AS variants was calculated by a weighted method (see Materials and Methods). Although the mean AS variants were slightly lower than those calculated with an un-weighted method (Figure 2), the positive relationship between the number of AS variants and the number of APs remained. Genes with more APs showed an increased mean AS variant. Error bar, 95% confidence intervals obtained from nonparametric bootstrapping. (B) The mean number of AS variants for each AP group of mouse APAS genes. The number of AS variants was calculated by a weighted method (see Material and Methods). Similar to what is observed in human genes, genes with more APs had an increased mean number of AS variants. Error bar, 95% confidence intervals obtained from nonparametric bootstrapping.
(0.29 MB TIF)

Figure S2 Number of APs is positively correlated with number of exons per gene. Genes with more APs have notably more exons in human (A) and mouse (B). The gene number information was extracted from AltSplice-rel3.splice-patterns.txt in the AltSplice database. Spearman ranked correlation coefficients R = 0.27 for human genes, and R = 0.20 for mouse genes.
(0.34 MB TIF)

Figure S3 The influence of gene exon number on the correlation between alternative promoter number and alternative splicing number. In order to verify that our results are not due to the correlation of AP with the number of exons, we examined the correlation for specific intervals of exon count (2∼3 exons, 4∼5 exons, 6∼7exons, 8∼exons). As shown in figure below, positive correlations were preserved across all the ranges for both human (A) and mouse genes (B).
(0.39 MB TIF)

Figure S4 AP number has no correlation with EST coverage per exon. The number of APs is independent of the number of transcripts per exon for human genes (A) and mouse genes (B), respectively. The number of transcripts covering a given gene was extracted from the AltSplice database. Spearman ranked correlation coefficients R = −0.022 for human gene and R = −0.036 for mouse genes.
(0.32 MB TIF)

Figure S5 Percentage distributions of “AS-TPs”, “Non-AS-TPs” and “C5T-TPs” across different TSS distance intervals.” The intervals of TSS distance used to plot the figures are: 0∼50 bp (A), 50∼150 bp (B), 150∼300 bp (C), 300∼500 bp (D), 500∼1000 bp (E), 1000∼bp (F). The figures show that the distribution bias toward “Non-AS-TPs” is preserved across all spans in which TSS distances are <300 bp (A, B and C), whereas distribution bias towards “AS-TPs” can be observed across all other spans in which TSS distances are >300 bp (D, E and F).
(0.66 MB TIF)

Figure S6 Multiple-promoter genes tend to be expressed more broadly than those with single promoters. Box-plot of the number of tissues for all genes (All), single-promoter genes (Single-promoter) and multiple-promoter genes (Multiple-promoter).The thick black line indicates the median tissue number for each gene category. The median number of tissues for multiple-promoter genes (13) is statistically larger than that for the single-promoter genes (7) (Wilcoxon Test, p<0.001). Microarray gene expression data for 79 human tissues produced by Su et al., 2004 was downloaded from Gene Expression Omnibus (GEO) (http://www.ncbi.nlm.nih.gov/geo) and processed using the Bioconductor affy package (http://www.bioconductor.org/).
(0.22 MB TIF)
