# Supplementary figures and images for: Alternative Promoters Influence Alternative Splicing at the Genomic Level
Source: PLoS One. 2008 Jun 18;3(6):e2377. doi: 10.1371/journal.pone.0002377 (PMC2409967; doi:10.1371/journal.pone.0002377)

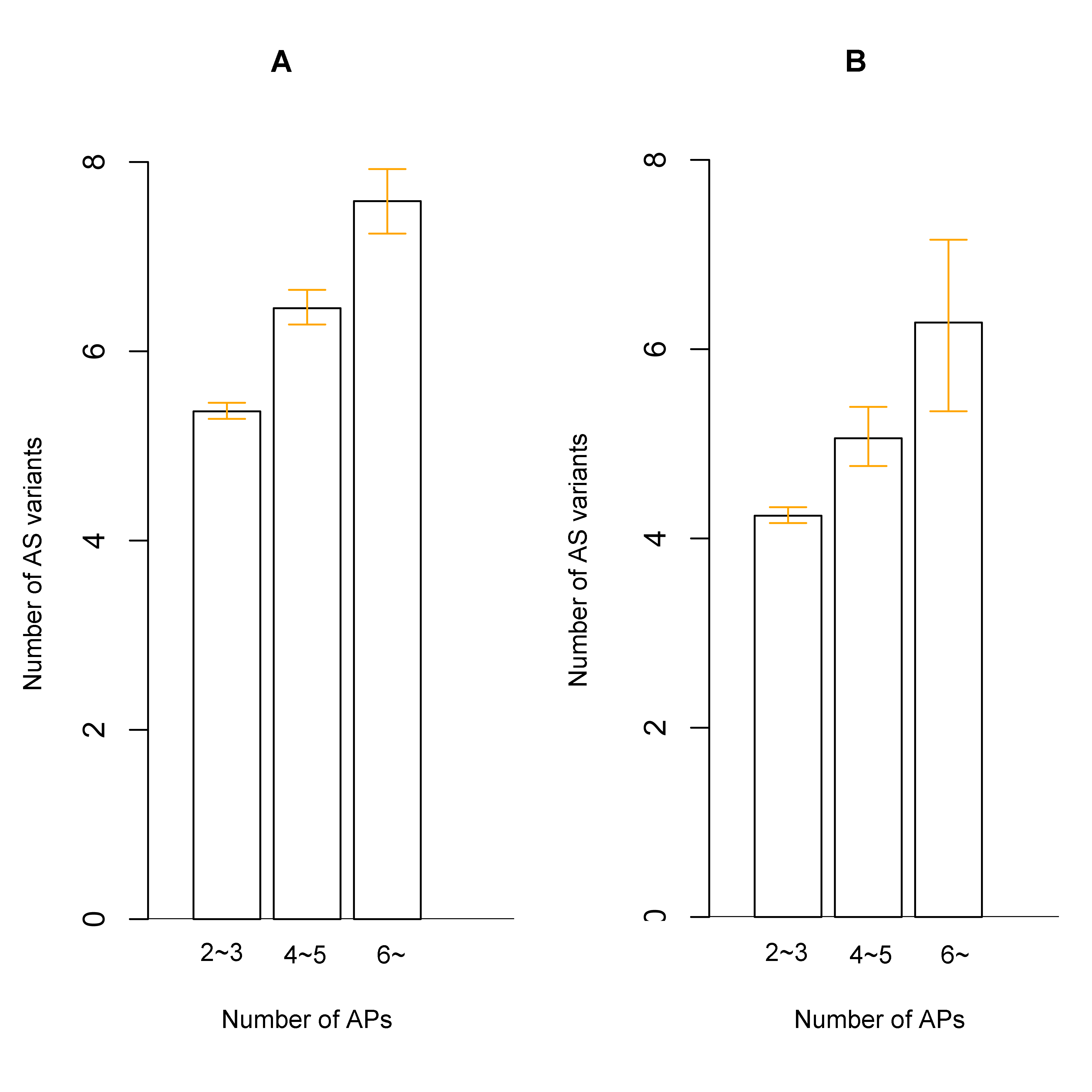

Supplement: Figure S1 — Positive relationship between the number of APs and the number of AS variants calculated by a weighted method in APAS genes. (A) The mean number of AS variants for each AP group of human APAS genes. The number of AS variants was calculated by a weighted method (see Materials and Methods). Although the mean AS variants were slightly lower than those calculated with an un-weighted method (Figure 2), the positive relationship between the number of AS variants and the number of APs remained. Genes with more APs showed an increased mean AS variant. Error bar, 95% confidence intervals obtained from nonparametric bootstrapping. (B) The mean number of AS variants for each AP group of mouse APAS genes. The number of AS variants was calculated by a weighted method (see Material and Methods). Similar to what is observed in human genes, genes with more APs had an increased mean number of AS variants. Error bar, 95% confidence intervals obtained from nonparametric bootstrapping. (0.29 MB TIF) [file pone.0002377.s001.tif]

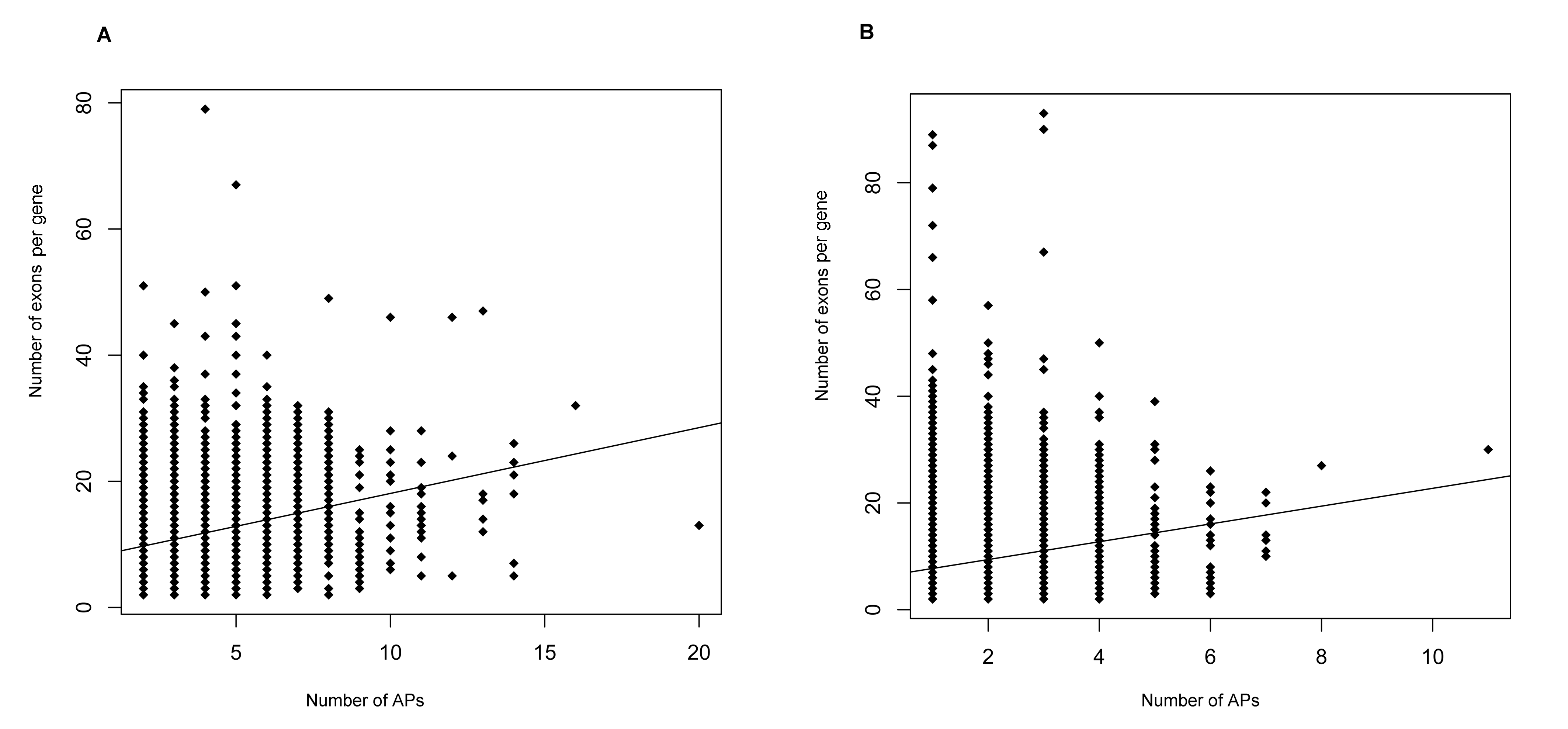

Supplement: Figure S2 — Number of APs is positively correlated with number of exons per gene. Genes with more APs have notably more exons in human (A) and mouse (B). The gene number information was extracted from AltSplice-rel3.splice-patterns.txt in the AltSplice database. Spearman ranked correlation coefficients R = 0.27 for human genes, and R = 0.20 for mouse genes. (0.34 MB TIF) [file pone.0002377.s002.tif]

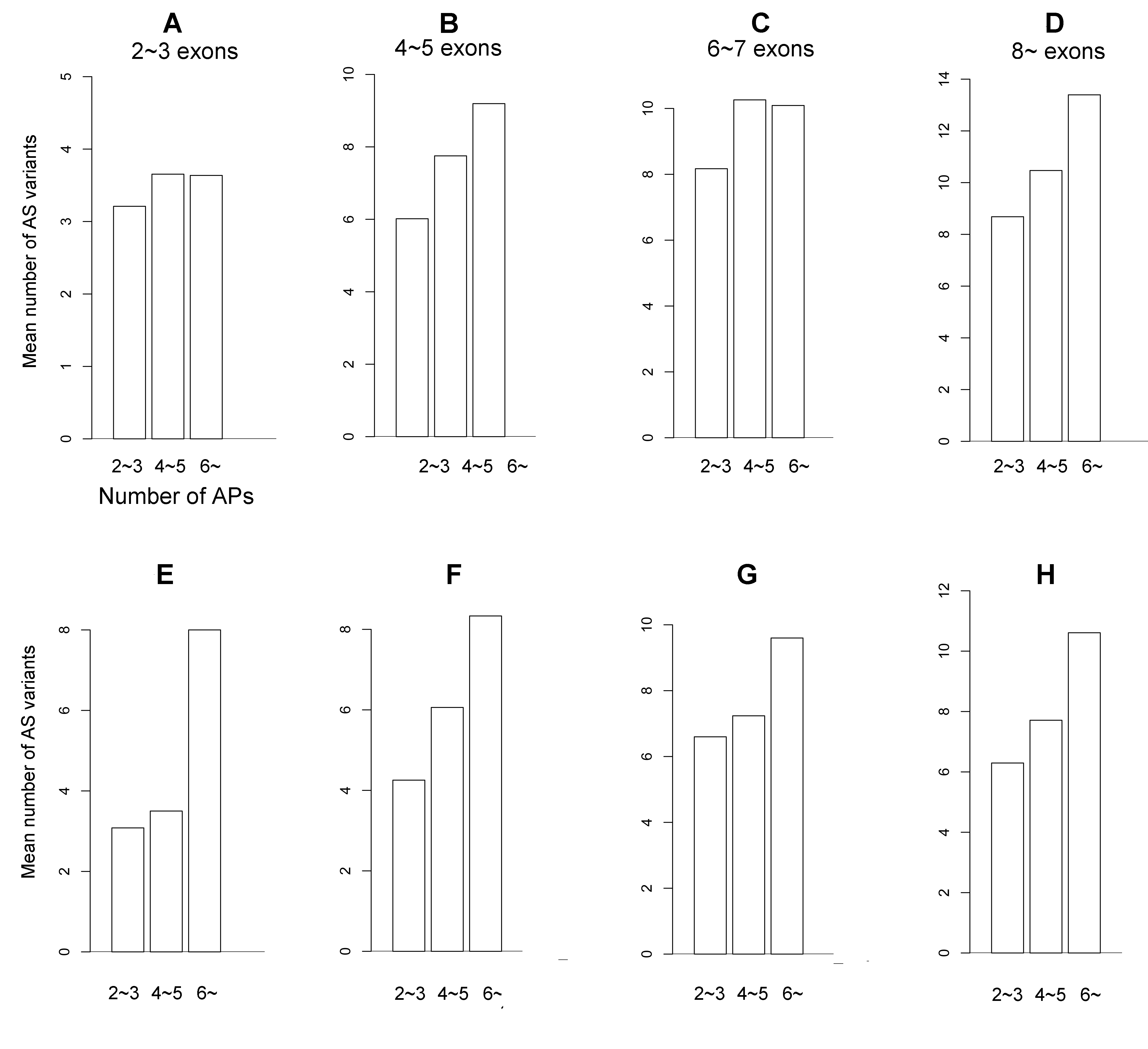

Supplement: Figure S3 — The influence of gene exon number on the correlation between alternative promoter number and alternative splicing number. In order to verify that our results are not due to the correlation of AP with the number of exons, we examined the correlation for specific intervals of exon count (2∼3 exons, 4∼5 exons, 6∼7exons, 8∼exons). As shown in figure below, positive correlations were preserved across all the ranges for both human (A) and mouse genes (B). (0.39 MB TIF) [file pone.0002377.s003.tif]

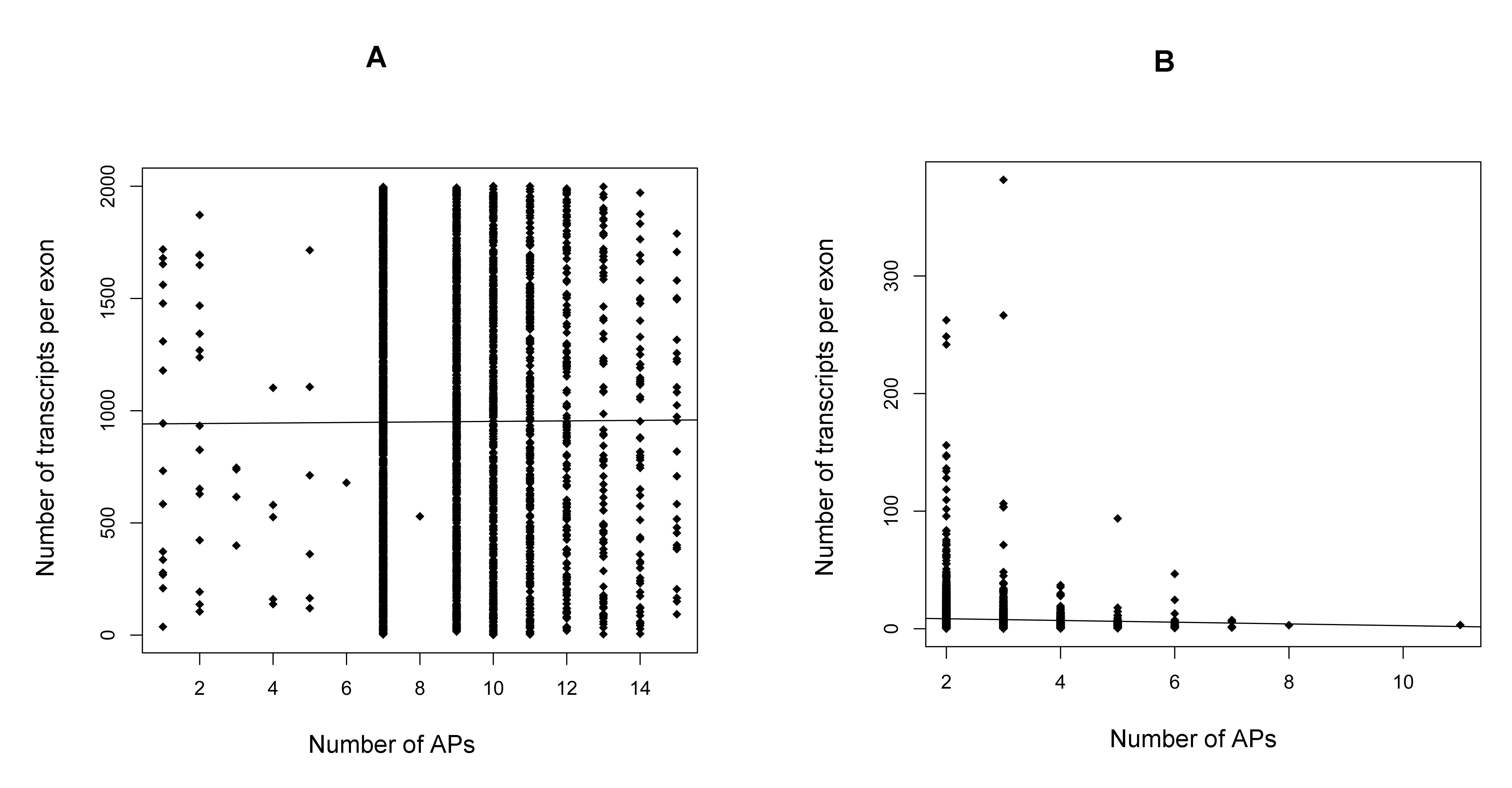

Supplement: Figure S4 — AP number has no correlation with EST coverage per exon. The number of APs is independent of the number of transcripts per exon for human genes (A) and mouse genes (B), respectively. The number of transcripts covering a given gene was extracted from the AltSplice database. Spearman ranked correlation coefficients R = −0.022 for human gene and R = −0.036 for mouse genes. (0.32 MB TIF) [file pone.0002377.s004.tif]

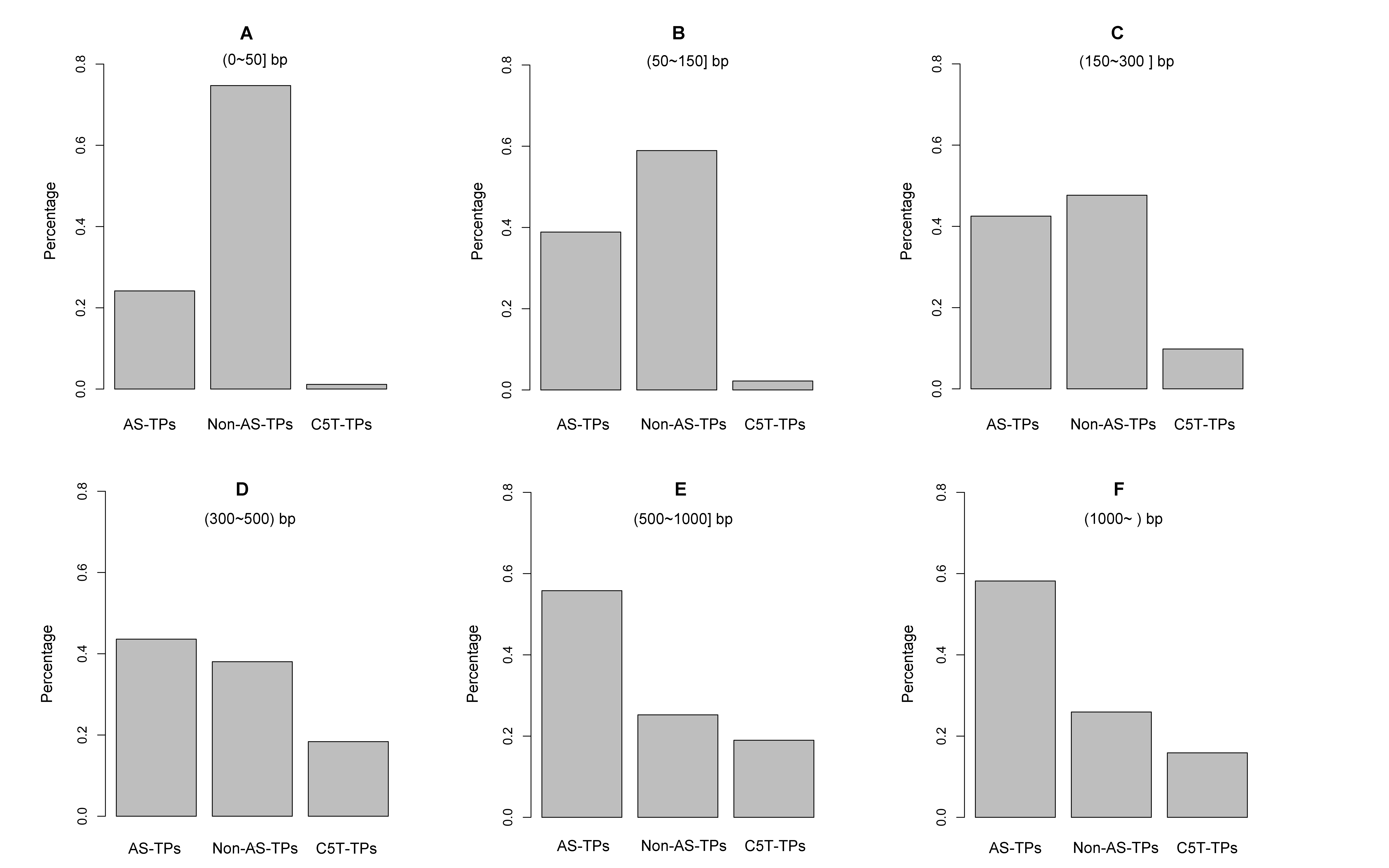

Supplement: Figure S5 — Percentage distributions of “AS-TPs”, “Non-AS-TPs” and “C5T-TPs” across different TSS distance intervals.” The intervals of TSS distance used to plot the figures are: 0∼50 bp (A), 50∼150 bp (B), 150∼300 bp (C), 300∼500 bp (D), 500∼1000 bp (E), 1000∼bp (F). The figures show that the distribution bias toward “Non-AS-TPs” is preserved across all spans in which TSS distances are <300 bp (A, B and C), whereas distribution bias towards “AS-TPs” can be observed across all other spans in which TSS distances are >300 bp (D, E and F). (0.66 MB TIF) [file pone.0002377.s005.tif]

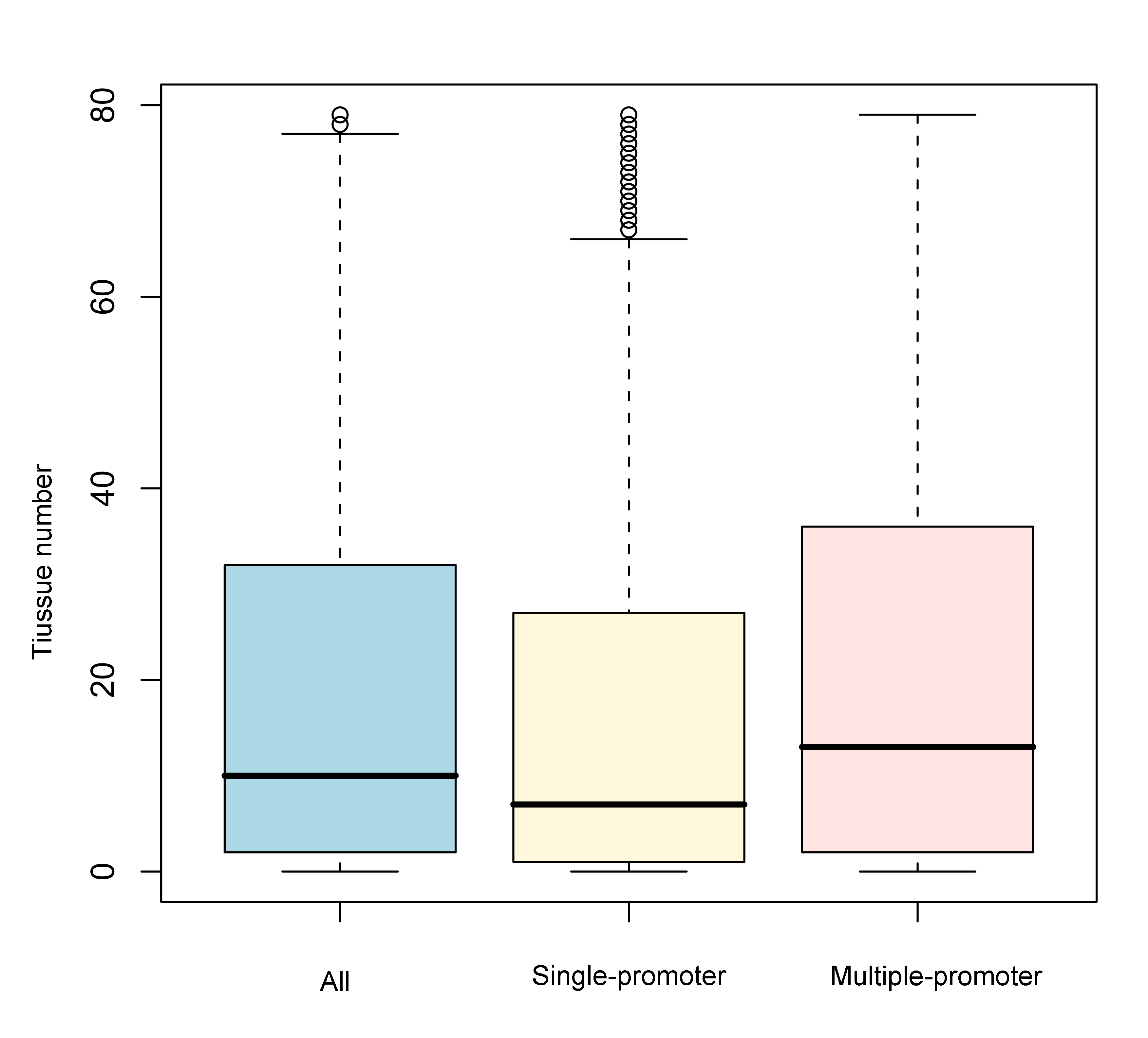

Supplement: Figure S6 — Multiple-promoter genes tend to be expressed more broadly than those with single promoters. Box-plot of the number of tissues for all genes (All), single-promoter genes (Single-promoter) and multiple-promoter genes (Multiple-promoter).The thick black line indicates the median tissue number for each gene category. The median number of tissues for multiple-promoter genes (13) is statistically larger than that for the single-promoter genes (7) (Wilcoxon Test, p<0.001). Microarray gene expression data for 79 human tissues produced by Su et al., 2004 was downloaded from Gene Expression Omnibus (GEO) (http://www.ncbi.nlm.nih.gov/geo) and processed using the Bioconductor affy package (http://www.bioconductor.org/). (0.22 MB TIF) [file pone.0002377.s006.tif]
